# Acetyl coenzyme A kinetic studies on *N*-acetylation of environmental carcinogens by human *N*-acetyltransferase 1 and its NAT1*14B variant

**DOI:** 10.3389/fphar.2022.931323

**Published:** 2022-10-28

**Authors:** Mariam R. Habil, Mark A. Doll, David W. Hein

**Affiliations:** Department of Pharmacology & Toxicology, University of Louisville School of Medicine, Louisville, KY, United States

**Keywords:** arylamine N-acetyltransferase 1, acetyl coenzyme A, 4-aminobiphenyl, β-naphthylamine, benzidine, 3, 4-dimethylaniline

## Abstract

N-acetyltransferase 1 (NAT1) is a xenobiotic metabolizing enzyme that uses acetyl coenzyme A (AcCoA) as a cofactor for *N*-acetylation of many carcinogens including aromatic amines and alkylanilines. NAT1 is characterized by single nucleotide polymorphisms (SNPs) that may modulate affinity towards AcCoA. In the current study, we used Chinese hamster ovary (CHO) cells stably transfected with human NAT1*4 (reference allele) or NAT1*14B (variant allele) to measure AcCoA kinetic parameters for *N*-acetyltransferase activity measurements towards *p*-aminobenzoic acid (PABA), 4-aminobiphenyl (4-ABP), β-naphthylamine (BNA), benzidine and 3,4-dimethylaniline (3,4-DMA). Our results showed higher *N*-acetylation rates for each substrate catalyzed by NAT1*4 compared to NAT1*14B. NAT1*4 exhibited higher affinity to AcCoA when catalyzing the *N*-acetylation of BNA and benzidine compared to NAT1*14B. The results of the current study provide further insights into differences in carcinogen metabolism among individuals possessing the NAT1*14B haplotype.

## Introduction

Arylamine *N*-acetyltransferases (NATs) are xenobiotic metabolizing enzymes which play important roles in the metabolism and detoxification of many drugs and carcinogens (Walraven et al., 2008b). Two NATs (*N*-acetyltransferase 1; NAT1 and N-acetyltransferase 2; NAT2) have been characterized in humans with similar structure (Sim et al., 2008). However, they exhibit different physiological roles and substrate affinities (Sim et al., 2003; Doll et al., 2010). NAT1 shows substrate specificity for *p*-aminobenzoic acid (PABA), while NAT2 shows substrate specificity for sulfamethazine (Hein, 2006).

NAT1 is a polymorphic enzyme due to single nucleotide polymorphisms or SNPs associated with decreased enzyme activity and changes in protein stability (Butcher et al., 2002; Hein, 2009; Zhou et al., 2013; Hein et al., 2018). The most common NAT1 variant allele associated with reduced acetylator phenotype is NAT1*14B. It is characterized by a single nucleotide polymorphism G560A (rs4986782) resulting in an amino acid substitution R187Q (Walraven et al., 2008a). NAT1*14B has been associated with an increased risk of smoking induced lung cancer (Bouchardy et al., 1998). Also, a recent study reported that individuals carrying NAT1*14B developed urinary bladder cancer with higher muscle-invasiveness and higher tumor grade compared to individuals with reference NAT1*4 suggesting the importance of NAT1*14B in cancer risk studies (El Kawak et al., 2020).

NATs utilize acetyl coenzyme A (AcCoA) as a cofactor to catalyze *N*- (usually deactivation) or *O*-acetylation (usually activation) of aromatic amines and heterocyclic amine pro-carcinogens (Sim et al., 2008). First, AcCoA binds to NAT1 leading to formation of the acetylated enzyme. Then, aromatic amines bind to the acetylated enzyme that eventually leads to formation of the acetylated product and release of CoA in a ping-pong bi-bi reaction mechanism (Sinclair et al., 2000; Walraven et al., 2008a; Tsirka et al., 2018).

Aromatic amines undergo hepatic hydroxylation catalyzed by CYP1A2 (Bartsch et al., 2000). *O*-acetylation of these hydroxylated aromatic amines by NAT1 leads to formation of reactive electrophiles that covalently bind to nucleophilic sites on proteins, DNA and RNA, forming adducts potentially leading to mutagenesis and carcinogenesis (Barnes et al., 2018; Leggett et al., 2022).

4-aminobiphenyl (4-ABP), beta-naphthylamine (BNA) and benzidine are aromatic amine carcinogens found in cigarette smoke and hair dyes (Chung, 2013). 3,4-Dimethylaniline is a monocyclic amine from the group of alkylanilines that have been used as dyestuffs and pharmaceutical intermediates and can be found in tobacco smoke (Skipper et al., 2010). Both aromatic amines and alkylanilines have been linked to urinary bladder cancer (Gan et al., 2004; Letasiova et al., 2012). Previous studies have shown the role of NAT1 genetic polymorphism in *N*-acetylation rates for arylamine carcinogens such as 4-ABP (Millner et al., 2012b).

Aromatic amines 4-ABP and BNA exhibit higher affinity for NAT2 compared to NAT1 whereas the opposite was observed for benzidine and 3,4-DMA (Leggett et al., 2021). AcCoA is a key regulator of several cellular processes including enzyme activity (Cai and Tu, 2011; Bradshaw, 2021). Its concentration is subject to fluctuation upon variations in its synthesis and use. Moreover, it can be affected by the fed and the metabolic states of cells (Pietrocola et al., 2015; Shi and Tu, 2015). NAT1 catalyzes the hydrolysis of AcCoA in a folate-dependent manner (Laurieri et al., 2014; Stepp et al., 2015). Also, MDA-MB-231 and MCF-7 NAT1 knockout human breast cancer cell lines exhibit increased AcCoA levels relative to their respective parental cell lines (Stepp et al., 2019). Previous studies showed that AcCoA affects protein acetylation and acetylation of NAT1 enhances its activity as it prevents its proteasomal degradation (Butcher et al., 2004; Bradshaw, 2021). However, the kinetics of AcCoA and its effect on *N*-acetylation of environmental carcinogens need to be investigated. The aim of this study is to compare AcCoA kinetics between human NAT1*4 and the NAT1*14B variant expressed in mammalian cell cultures.

## Materials and methods

**Chemicals.** Acetyl-CoA, para-aminobenzoic acid (PABA), *N*-acetyl PABA, 4-aminobiphenyl (4-ABP), *N*-acetyl ABP, β-naphthylamine (BNA), *N*-acetyl BNA, benzidine and 3,4-dimethylaniline (3,4-DMA) were purchased from Sigma Aldrich. *N*-acetyl-benzidine or *N*-(4'-amino-[1,1'-biphenyl]-4-yl) acetamide was purchased from Alinda Chemical (Moscow, Russia), *N*-acetyl 3,4 DMA or *N*-(3,4-dimethylphenyl) acetamide was purchased from BIONET/Key Organics Ltd. (Cornwall, United Kingdom).

**Chinese Hamster Ovary (CHO) Cells.** UV5-CHO cells that express human *CYP1A2* and NAT1*4 or NAT1*14B were constructed and characterized as previously described (Millner et al., 2012a; Millner et al., 2012b). UV5/CHO cells, a nuclease excision repair-deficient derivative of AA8 that are hypersensitive to bulky DNA lesions, were obtained from the American Type Culture Collection. Cells were grown in alpha-modified minimal essential medium (Cambrex) without L-glutamine, ribosides, and deoxyribosides supplemented with 10% fetal bovine serum (Hyclone), 100 units/ml penicillin, 100 μg/ml streptomycin (Cambrex), and 2 mM L-glutamine (Cambrex) at 37°C in 5% CO_2_. The cells used in this study were previously stably transfected with a single FRT integration site. The FRT site allowed stable transfections to use the Flp-In System (Invitrogen). The FRT site allows recombination to occur immediately downstream of the hygromycin resistance gene, allowing for hygromycin selectivity only after Flp-recombinase-mediated integration. The UV5/FRT cells were further modified by stable integration of human CYP1A2 and NADPH-cytochrome P450 reductase gene. These cells were expanded, and intact geneticin-resistant cells were assayed for CYP1A2 activity by measuring 7-ethoxyresorufin O-deethylase (EROD) activity as previously described (Bendaly et al., 2007). UV5/1A2 cells were stably transfected with pcDNA5/FRT containing NATb/NAT1*4 and NATb/NAT1*14B constructs using Effectene transfection reagent (QIAGEN, Valencia, CA) following the manufacturer’s recommendations. Because the pcDNA5/FRT vector contains a hygromycin resistance cassette, 120 g/ml hygromycin (Invitrogen) was added to media to select for cells containing the pcDNA5/FRT plasmid. The amount of NAT1 produced in UV5/*1A2* cells stably transfected with NAT1*4 or NAT1*14B was determined by Western blot (Millner et al., 2012b). The NAT1*4 and NAT2*14B-transfected cells were characterized for *N*-acetylation of PABA, a NAT1-selective substrate as described below. NAT1 haplotypes were determined by allele specific polymerase chain reaction as previously described (Doll and Hein, 2002). Quantitative RT-PCR (RT-qPCR) assays were used to assess the relative amount of CYP1A2 mRNA in cells in CHO cells and CYP1A2 protein expression was measured using in cell western protocol as previously described (Habil et al., 2022).

**Cell lysate preparation.** CHO cells were lysed in 20 mM sodium phosphate buffer (pH 7.4), 1 mM EDTA, 1 mM dithiothreitol, 100 μM phenylmethanesulfonyl fluoride, Pierce protease inhibitor minitablets (Thermo Scientific) plus 0.2% triton-x-100 (Sigma). Lysates were then placed on a rotator at 4°C for 10 min. Then lysates were centrifuged at 15,000 *g* at 4°C for 20 min and the supernatant was removed, aliquoted and assayed for enzymatic activity as described below.

***N*- acetyltransferase assays.** *In vitro N*-acetyltransferase assays were done by using cell lysates of UV5-CHO cells stably expressing CYP1A2 and either NAT1*4 or NAT1*14B. AcCoA kinetic constants were determined from assays conducted in the presence of 250 µM PABA, 4-ABP, BNA, benzidine or 3,4-DMA with varying concentrations (31.3–1,000 µM) of AcCoA. The reaction was terminated by the addition of 1/10 volume of 1 M acetic acid. Reaction tubes were centrifuged at 15,000 × g for 10 min to precipitate protein. PABA, 4-ABP, BNA, benzidine, 3,4-DMA and their *N*-acetylated products were separated and identified by high performance liquid chromatography (HPLC) following injection (40 µL) onto a 125 × 4 mm LiChrospher 100 RP-100 5 µM C18 HPLC column.

The amounts of PABA and acetyl-PABA produced was determined following separation and quantitation by HPLC subjected to a gradient of 96% 20 mM sodium perchlorate pH 2.5/4% acetonitrile to 88% 20 mM sodium perchlorate pH 2.5/12% acetonitrile over 7 min, then to 85% 20 mM sodium perchlorate pH 2.5/15% acetonitrile over 4 min. Retention times for PABA and acetyl-PABA were 2.5 and 8.8 min, respectively. The UV detector was set at 280 nm.

The amounts of acetyl-ABP and acetyl-BNA produced were determined following separation and quantitation by HPLC subjected to a gradient of 85% 20 mM sodium perchlorate pH 2.5/15% acetonitrile for 15 min to 65% 20 mM sodium perchlorate pH 2.5/35% acetonitrile over 2.5 min, then to 85% 20 mM sodium perchlorate pH 2.5/15% acetonitrile over 2.5 min. Retention times for 4-ABP and acetyl-ABP were 9.5 and 12.5 respectively and for BNA and acetyl-BNA, retention times were 3.97 and 10.1 min, respectively. The UV detector was set at 260 nm.

For benzidine and 3,4-DMA, the amounts of benzidine and 3,4-DMA and their *N*-acetylated products produced were determined following separation and quantitation by HPLC subjected to a gradient of 100% 20 mM sodium perchlorate pH 2.5/0% acetonitrile for 5 min to 0% 20 mM sodium perchlorate pH 2.5/100% acetonitrile over 10 min, then to 100% 20 mM sodium perchlorate pH 2.5/0% acetonitrile over 5 min. Retention times for benzidine and acetyl-benzidine were 6.9 and 8.5 min respectively and for 3,4-DMA and acetyl-3,4-DMA were 8.08 and 10.1 respectively. The UV detector was set at 320 nm for benzidine and 250 nm for 3,4-DMA.

For all samples, protein concentrations of cell lysates were determined using the Bio-Rad protein assay kit (Bio-Rad, Richmond, CA) and activity was calculated in nmoles of acetylated product/ml/min/mg protein.

## Statistical analysis

Kinetic parameters apparent AcCoA *K*m and *V*max were calculated using the Michaelis- Menten equation. Differences in apparent AcCoA *K*m and *V*max values were tested for significance by unpaired student *t*-test. To compare apparent *K*m and *V*max values for all substrates with the same allele, we used,one-way analysis of variance (ANOVA) followed by Tukey test or two-way analysis of variance (ANOVA) followed by Bonferroni test (Graph Pad Prism 9).

## Results

In this study, we used CHO mammalian cells which express human CYP1A2 and either human NAT1*4 or NAT1*14B. The NAT1*14B variant is characterized by single nucleotide polymorphism (SNP) at G560A (R187Q) leading to change in the active site of the enzyme that affect affinity towards PABA (Zhu and Hein, 2008).

**FIGURE 1:**
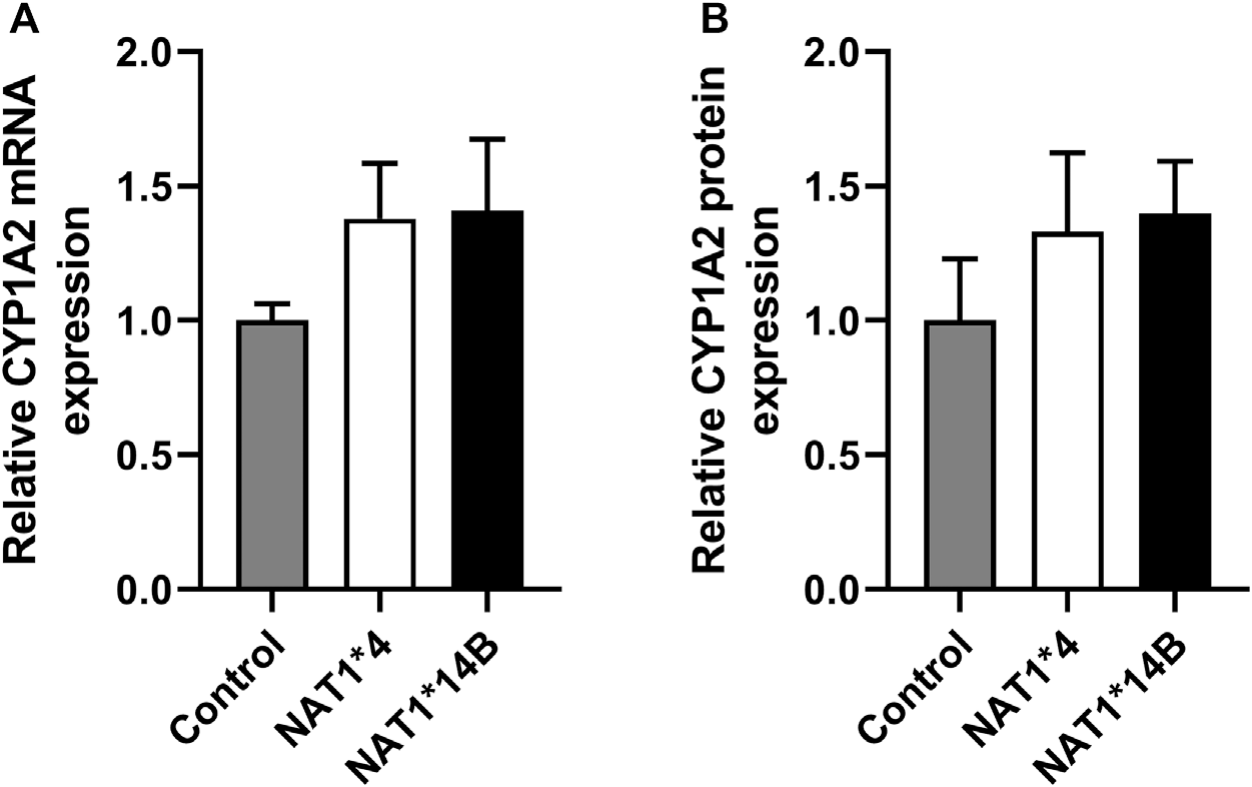
CYP1A2 **(A)** mRNA and **(B)** protein expression. CYP1A2 mRNA and protein levels were higher in CYP1A2-transfected compared to non-transfected (control) CHO cells and did not differ significantly among the CHO cells transfected with CYP1A2 and NAT1*4 or NAT1*14B. (*p* > 0.05). Statistical significance was determined using one-way ANOVA followed by a Tukey’s ’s post-hoc test. Data illustrates mean ± SEM of four replicates.

**FIGURE 2:**
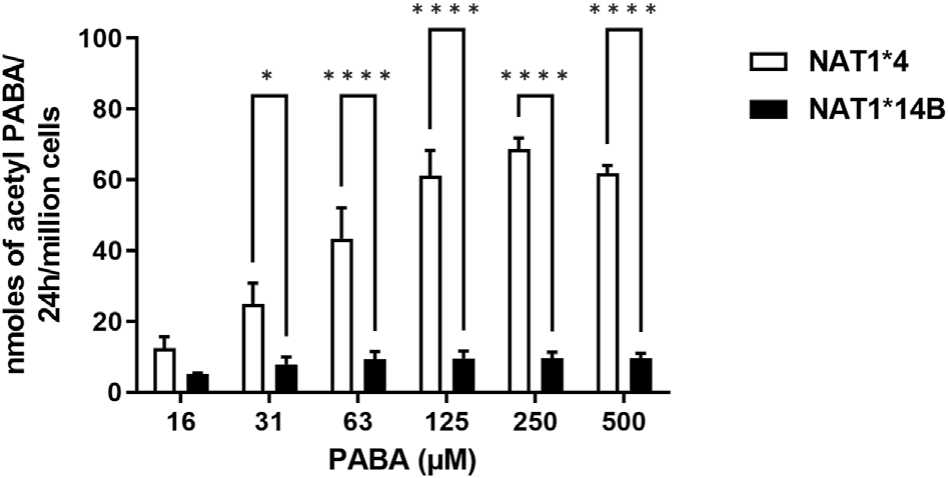
*N*-acetylation rate of PABA *in situ* in CHO cell lines. Statistical significance was determined using two-way ANOVA followed by a Bonferroni’s post-hoc test. Data illustrates mean ± SEM of three independent experiments (*p < 0.05, ****p < 0.001).

First, we validated CHO cells and our results showed that CYP1A2 mRNA and protein levels were elevated in CYP1A2-transfected compared to non-transfected (control) CHO cells and did not differ significantly between the CHO cells transfected with CYP1A2 and NAT1*4 or NAT1*14B (Figures 1A,B). CHO cells containing NAT1*4 or NAT1*14B were confirmed by allele-specific amplification as previously described (Doll and Hein, 2002). *N*-acetylation of PABA was measured to confirm the NAT1 alleles, with higher *N*-acetylation activity in CHO cells expressing NAT1*4 (*p* < 0.0001) than in CHO cells expressing NAT1*14B (Figure 2).

**FIGURE 3:**
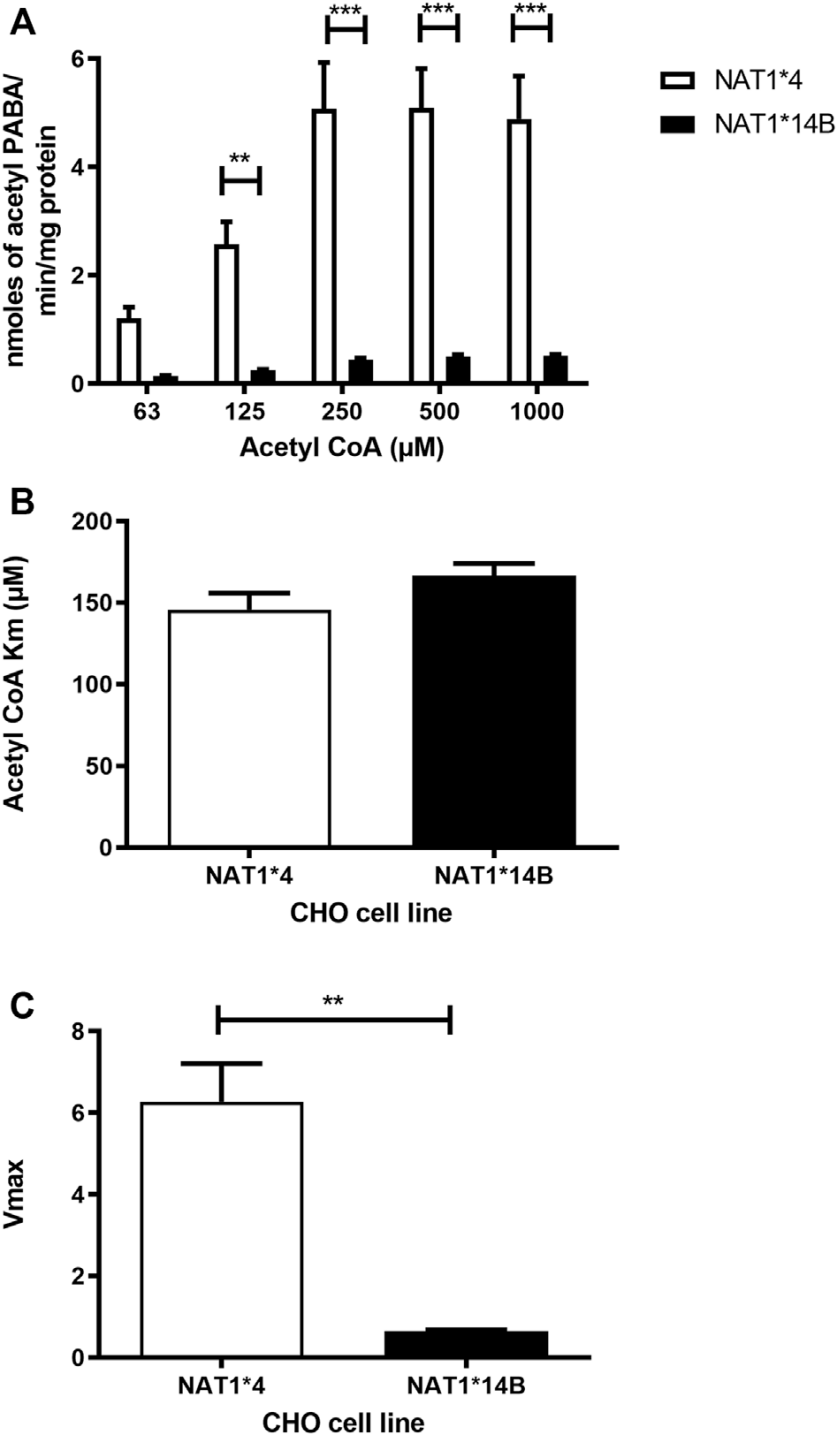
**(A)** Effect of NAT1 alleles on *in vitro N*-acetyltransferase catalytic activities towards PABA using AcCoA 62.5–1,000 µM. **(B)** Apparent AcCoA *K*m did not significantly differ between both alleles. **(C)** *V*max (nmol/min/mg) was higher in NAT1*4 compared to NAT1*14B. Data represents Mean ± S.E.M. of three separate experiments (**p < 0.01, ***p < 0.001).

**FIGURE 4:**
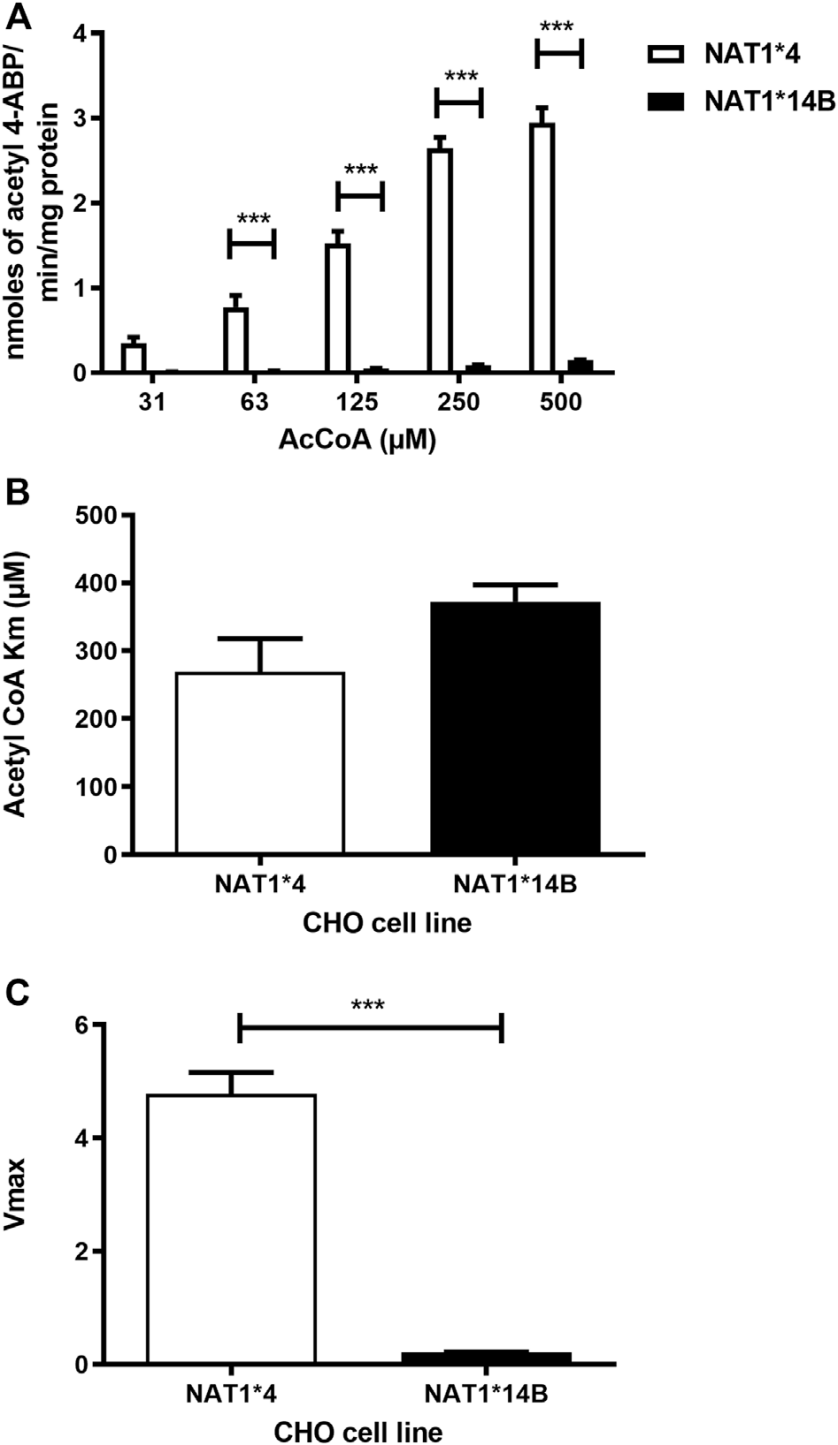
**(A)** Effect of NAT1 alleles on *in vitro N*-acetyltransferase catalytic activities towards 4-ABP using AcCoA A 31.3–500 µM. **(B)** Apparent AcCoA *K*m did not significantly differ between both alleles. **(C)** *V*max (nmol/min/mg) was significantly higher in NAT1*4 compared to NAT1*14B. Data represents Mean ± S.E.M. of three separate experiments (***p < 0.001).

**FIGURE 5:**
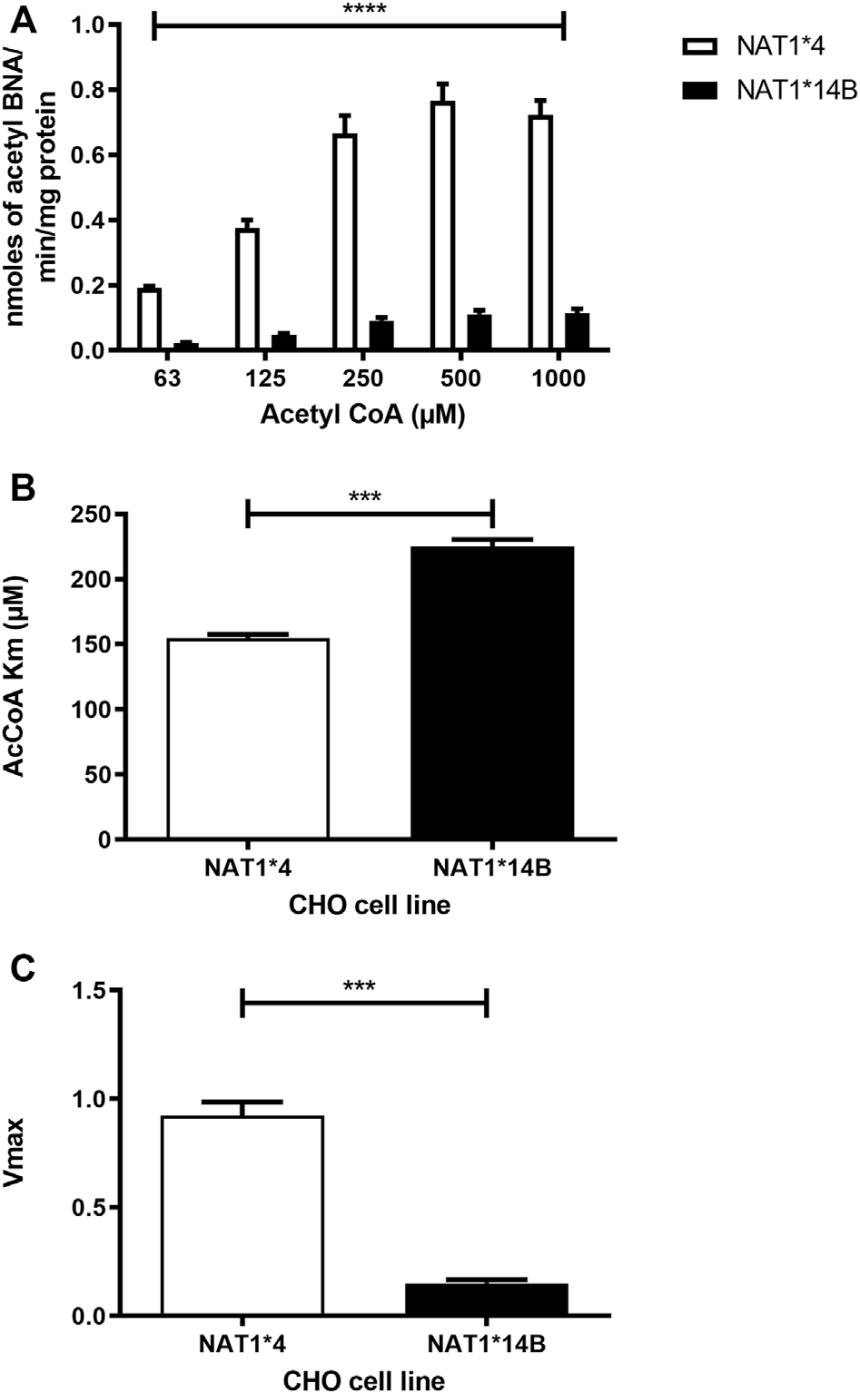
**(A)** Effect of NAT1 alleles on *in vitro N*-acetyltransferase catalytic activities towards BNA using AcCoA (62.5–1,000 µM). **(B)** Apparent AcCoA *K*m is higher in NAT1*14B compared to NAT1*4 (1.5- fold). **(C)** *V*max (nmol/min/mg) was significantly higher in NAT1*4 compared to NAT1*14B. Data represents Mean ± S.E.M. of three separate experiments (***p < 0.001, ****p < 0.0001).

**FIGURE 6:**
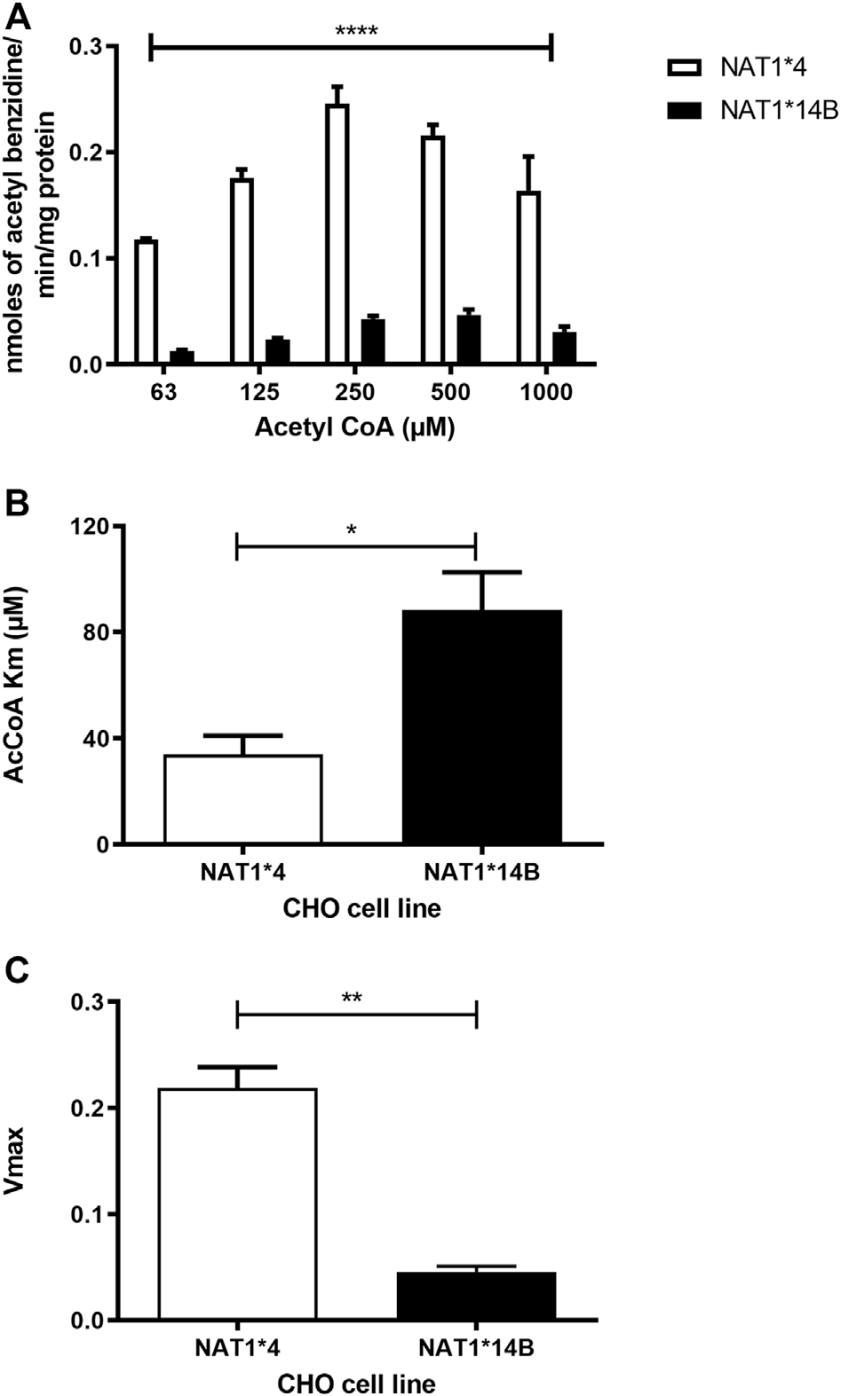
**(A)** Effect of NAT1 alleles on *in vitro N*-acetyltransferase catalytic activities towards benzidine using AcCoA (62.5–1,000 µM). **(B)** Apparent AcCoA *K*m is higher in NAT1*14B compared to NAT1*4 **(C)** *V*max (nmol/min/mg) was significantly higher in NAT1*4 compared to NAT1*14B. Data represents Mean ± S.E.M. of three separate experiments (*p < 0.05, **p < 0.01, ****p < 0.0001).

**FIGURE 7:**
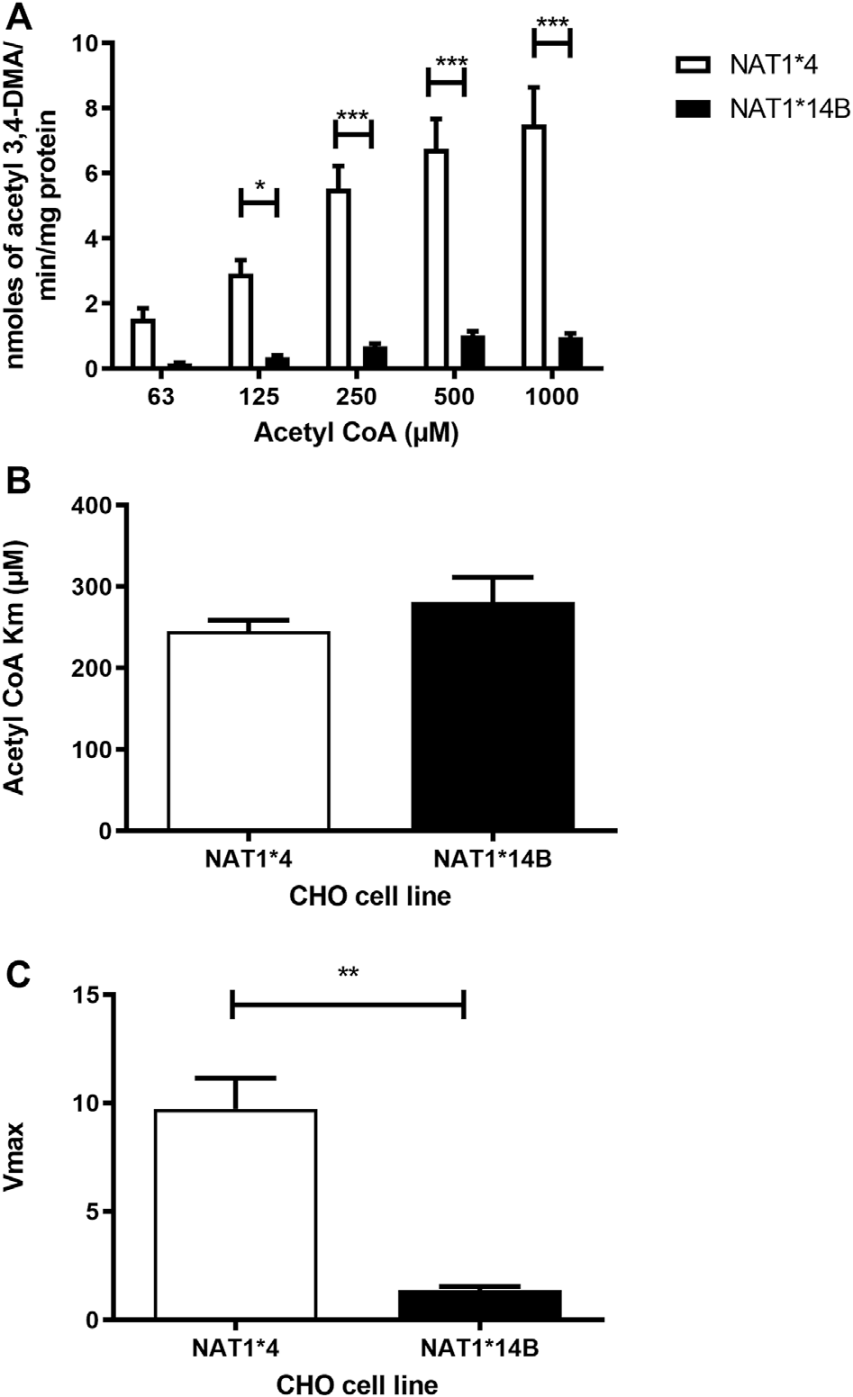
**(A)** Effect of NAT1 alleles on *in vitro N*-acetyltransferase catalytic activities towards 3,4-DMA using AcCoA (62.5–1,000 µM). **(B)** Apparent AcCoA *K*m did not significantly differ between both alleles. **(C)** *V*max (nmol/min/mg) was significantly higher in NAT1*4 compared to NAT1*14B. Data represents Mean ± S.E.M. of three separate experiments (*p < 0.05, **p < 0.01, ***p < 0.001).

Our results report that *in vitro N*-acetylation rates for all substrates were AcCoA concentration dependent. First, we used PABA which is the prototypic substrate for NAT1. NAT1*4 showed higher AcCoA apparent Vmax compared to NAT1*14B (*p* < 0.001) (Figure 3C). AcCoA apparent Km did not differ significantly between NAT1*4 and NAT1*14B. (Figure 3B). For 4-ABP, NAT1*4 had higher apparent AcCoA Vmax (Figure 4C) compared to NAT1*14B (*p* < 0.001). However, no significant difference was observed regarding their affinity towards AcCoA (Figure 4B). In the presence of BNA, NAT1*4 had higher affinity towards AcCoA compared to NAT1*14B (Figure 5B) reflected by the lower apparent *K*m (1.5- fold, *p* < 0.001). AcCoA Vmax was significantly higher in NAT1*4 compared to NAT1*14B (*p* < 0.001) (Figure 5C). AcCoA kinetic constants in the presence of benzidine showed similar pattern with apparent Vmax higher in NAT1*4 compared to NAT1*14B (Figure 6C) (*p* < 0.0001) and higher affinity towards AcCoA (apparent *K*m value in NAT1*4 was 2.6-fold lower than NAT1*14B (*p* < 0.05) (Figure 6B). AcCoA apparent Vmax in the presence of 3,4-DMA in NAT1*4 was higher in NAT1*4 compared to NAT1*14B (Figure 7C) (*p* < 0.001). However, AcCoA apparent *K*m values did not significantly differ (Figure 7B).

**FIGURE 8:**
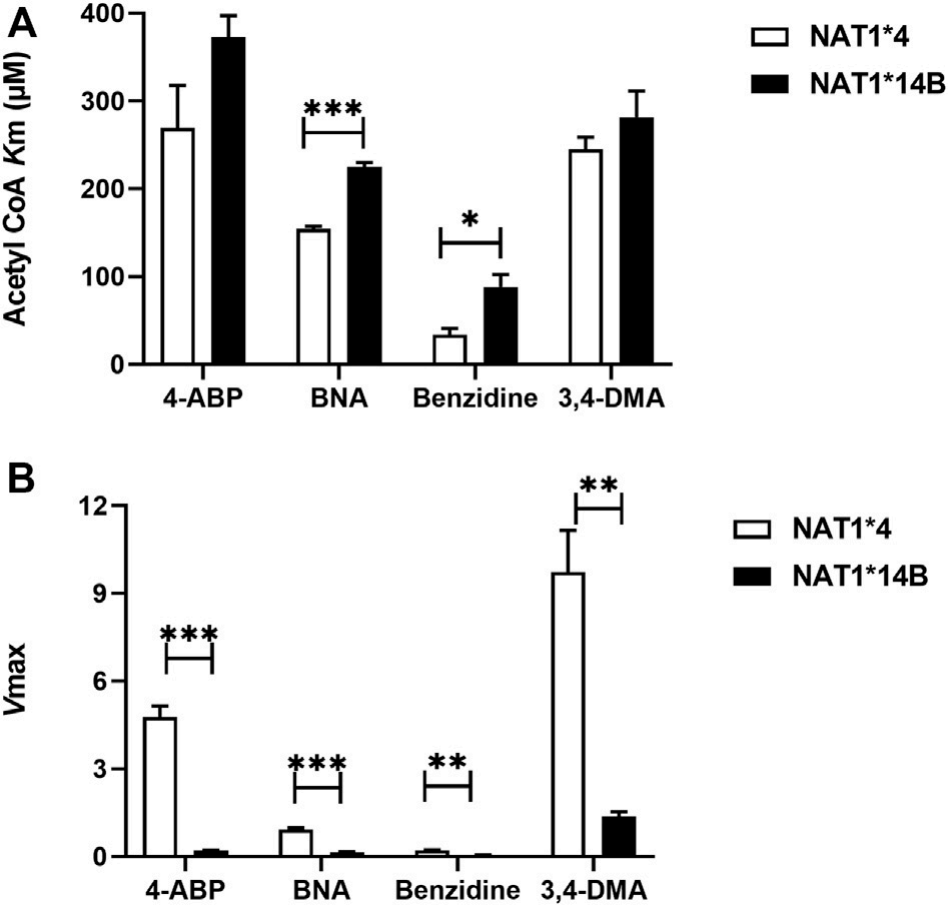
**(A)** Comparison of AcCoA *K*m values using different substrates for CHO cells expressing NAT1*4 or NAT1*14B **(B)** Comparison of *V*max values (nmol/min/mg) using different substrates for CHO cells expressing NAT1*4 or NAT1*14B. Data represents Mean ± S.E.M. of three separate experiments (*p < 0.05, **p < 0.01, ***p < 0.001).

Comparing AcCoA apparent Km values across substrates revealed that both NAT1*4 (*p* < 0.001) and NAT1*14B (*p* < 0.0001) have higher affinity to bind to AcCoA in presence of benzidine compared to the other carcinogens (Figure 8A). In contrast, the AcCoA apparent Vmax values across substrates were higher for NAT1*4 than NAT1*14B (Figure 8B).

## Discussion

The G560A (R187Q) SNP associated with NAT1*14B has been reported to modify apparent *K*m towards PABA and ABP, albeit in opposite directions for these two aromatic amines (Zhu and Hein, 2008) A previous study showed that the apparent *K*m of NAT1*14B was higher for PABA compared to NAT1*4 (Zhu and Hein, 2008), whereas the apparent *K*m of NAT1*14B was lower for 4-ABP and *N*-OH-ABP compared to NAT1*4 (Millner et al., 2012b). Also, Zhu and Hein compared AcCoA Km and there was no significant difference in AcCoA Km between NAT1*4 and NAT1*14B in presence of PABA (Zhu and Hein, 2008). Our current study confirmed similar results for AcCoA *K*m values using PABA as a substrate. Nevertheless, AcCoA kinetic constants in the presence of carcinogens showed that NAT1*4 exhibited higher affinity towards AcCoA compared to NAT1*14B using BNA and benzidine as substrates (*p* < 0.001 and *p* < 0.05 respectively).

NAT1*14B showed lower *V*_max_ than NAT1*4 for PABA, 4-ABP, and *N*-OH-ABP consistent with decreased ability of NAT1*14B to metabolize these substrates compared to NAT1*4 (Millner et al., 2012b). In the current study, we found similar results for *N*-acetylation of all carcinogens. Apparent *V*max for all substrates were higher in NAT1*4 compared to NAT1*14B. This is consistent with the effects of the G560A (R187Q) SNP in COS-1 cells (Doll and Hein, 2022).

Previous studies have shown that the intracellular acetyl CoA level can be affected by many factors including the fasting and the fed states and this in turn can affect acetylation of many proteins including enzymes (Xiong and Guan, 2012; Carrer et al., 2017). Our study showed that NAT1*4 has higher affinity for AcCoA compared to NAT1*14B. This may allow NAT1*4 to be in a more acetylated state consistent with higher *N*-acetylation activity.

In addition, comparison of AcCoA *K*m values within the same allele across different substrates revealed that NAT1*4 and NAT1*14B exhibited higher affinity to AcCoA in presence of benzidine compared to other substrates as reflected by the lower apparent AcCoA *K*m. This suggests the affinity towards AcCoA is dependent on the carcinogen substrate and is higher towards benzidine than other substrates. The *N*-acetylation of benzidine likely leads to its activation (Degen et al., 2004; Carreon et al., 2006).

**FIGURE 9:**
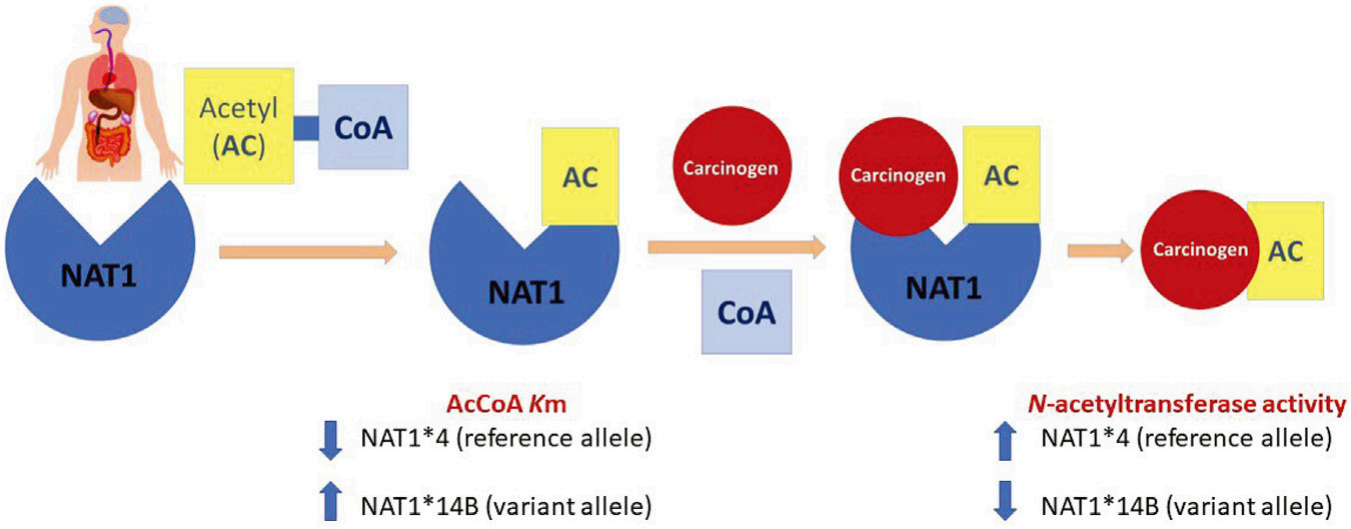
*N*-acetylation of carcinogens via NAT1 acts through a ping-pong bi-bi mechanism that requires two steps. First, AcCoA binds to the enzyme to form the acetylated form which is active. Then, binding of the carcinogen is followed by the release of the acetylated product. Our findings showed that NAT1*4 reference allele has higher *N*-acetylation compared to the variant allele NAT1*14B. Also, affinity of NAT1*4 towards AcCoA was higher than that of NAT1*14B represented by lower Km values.

A summary of the results of this study is illustrated in Figure 9. *In vitro* *N*-acetylation rate towards all substrates was higher in CHO cells expressing NAT1*4 than NAT1*14B (as shown in Figures 3A, 4A, 5A, 6A, 7A). Strengths of this study included that we have used a model stably transfected with human CYP1A2 and NAT1 alleles and used it to study human carcinogen metabolism. Also, we have included NAT1*14B allele which is an important variant associated with lung and urinary bladder cancer. In addition, we have developed HPLC methods to measure the N-acetylation of aromatic amines and alkylanilines that can be optimized to be used in human serum or urine samples. Furthermore, our study investigated the effect of NAT1 allelic variation on the affinity towards AcCoA which is an important regulatory cofactor for many cellular processes. On the other hand, some limitations were that we have compared only two NAT1 alleles for NAT1 enzyme. Also, we did not use human cell lines. Further studies should include other NAT1 allelic variants and use of human cell lines.

## Data Availability

The original contributions presented in the study are included in the article/Supplementary Material, further inquiries can be directed to the corresponding author.
